# Bistable Percepts in the Brain: fMRI Contrasts Monocular Pattern Rivalry and Binocular Rivalry

**DOI:** 10.1371/journal.pone.0020367

**Published:** 2011-05-23

**Authors:** Athena Buckthought, Samuel Jessula, Janine D. Mendola

**Affiliations:** McGill Vision Research Unit, Department of Ophthalmology, McGill University, Montreal, Quebec, Canada; University of Regensburg, Germany

## Abstract

The neural correlates of binocular rivalry have been actively debated in recent years, and are of considerable interest as they may shed light on mechanisms of conscious awareness. In a related phenomenon, monocular rivalry, a composite image is shown to both eyes. The subject experiences perceptual alternations in which the two stimulus components alternate in clarity or salience. The experience is similar to perceptual alternations in binocular rivalry, although the reduction in visibility of the suppressed component is greater for binocular rivalry, especially at higher stimulus contrasts. We used fMRI at 3T to image activity in visual cortex while subjects perceived either monocular or binocular rivalry, or a matched non-rivalrous control condition. The stimulus patterns were left/right oblique gratings with the luminance contrast set at 9%, 18% or 36%. Compared to a blank screen, both binocular and monocular rivalry showed a U-shaped function of activation as a function of stimulus contrast, i.e. higher activity for most areas at 9% and 36%. The sites of cortical activation for monocular rivalry included occipital pole (V1, V2, V3), ventral temporal, and superior parietal cortex. The additional areas for binocular rivalry included lateral occipital regions, as well as inferior parietal cortex close to the temporoparietal junction (TPJ). In particular, higher-tier areas MT+ and V3A were more active for binocular than monocular rivalry for all contrasts. In comparison, activation in V2 and V3 was reduced for binocular compared to monocular rivalry at the higher contrasts that evoked stronger binocular perceptual suppression, indicating that the effects of suppression are not limited to interocular suppression in V1.

## Introduction

Multistable images comprise important examples of conscious visual perceptual changes without any change in the stimulus being viewed. Multistability can be induced by either using an ambiguous figure with more than one perceptual interpretation such as the Necker cube [1] or Rubin's vase/face [2] or by showing different images to the left and right eye, as in binocular rivalry [3]. Other examples of multistability include the kinetic depth effect [4], multistable apparent motion [5], and ambiguous plaid motion [6].

Binocular rivalry has been extensively studied in psychophysical and fMRI paradigms. It is generally believed to involve competition between visual representations at multiple levels of the visual pathway [7]–[11]. Most previous functional neuroimaging studies of binocular rivalry reported activation in early visual areas (V1, V2, V3) [3], [12], [13], and further studies indicate that eye-specific dominance and suppression are reflected at an even earlier stage of visual processing, in the lateral geniculate nucleus [14], [15]. Traditional models of binocular rivalry have focused on interocular competition in area V1 where monocular neurons are known to exist [7], [8], [10], [11], or asynchrony of responses of binocular neurons in V1 [16]. Neuronal asynchrony in V1 might produce rivalrous response suppression at later stages in the visual pathway [16]. Indeed, other cortical regions are also implicated, such as occipito-parietal areas (V3a, V4d-topo, V7) [17], ventral temporal areas [18], [19], superior parietal lobe and caudal intraparietal sulcus [17], [19], [20], as well as frontal areas [19], [20].

**Figure 1:**
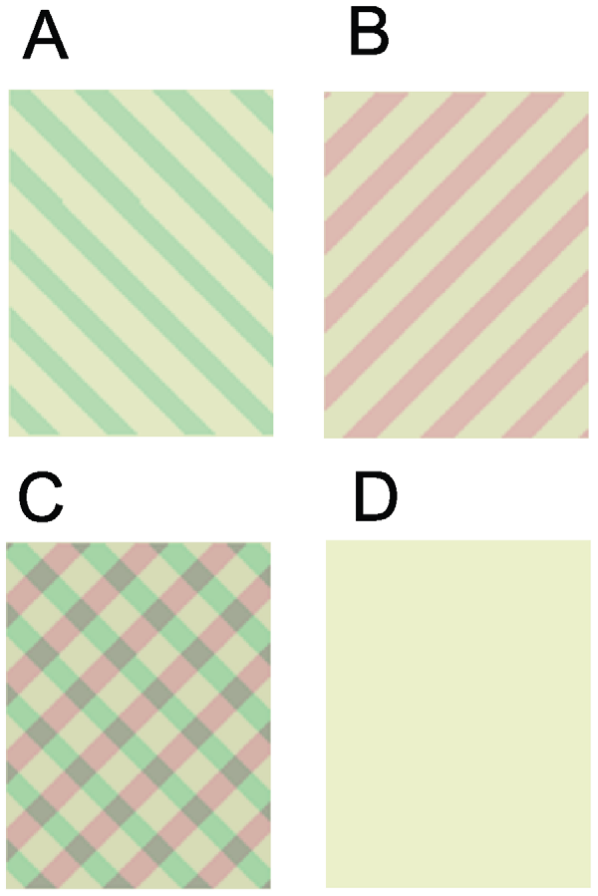
Stimuli used in the fMRI and psychophysics experiments. (A–B) Left and right oblique gratings used for dichoptic presentation in binocular rivalry. (C) Composite grating stimulus presented to both eyes for monocular rivalry. (D) Baseline blank condition.

In monocular rivalry, the subject experiences similar alternations between different perceptual representations of the same image [21]. The perceptual alternations are described as changes in clarity or salience of one of the two stimulus components in the composite image. This differs from the near complete reduction in visibility that accompanies suppression in binocular rivalry (Figure 1) [22]–[24]. A direct comparison of monocular rivalry and binocular rivalry is attractive as the same images with matched retinal stimulation can be used for both forms of bistability in order to isolate the effect of suppression in particular, and to determine if they share common neural mechanisms in general.

Current models of binocular rivalry include competition at multiple levels of the visual hierarchy. At the lowest level, binocular rivalry involves interocular inhibition between monocular neurons in V1 or the lateral geniculate nucleus (eye-based rivalry) [7], [8], [10], [11], [14], [15], [16]. At higher levels, binocular rivalry also involves competition between the representations of different patterns, which may be combined across the eyes (stimulus-based rivalry) [18], [24]–[26]. In principle, if monocular rivalry shares this high-level stimulus competition with binocular rivalry, but lacks the lower-level interocular competition, then this might explain the lower degree of perceptual suppression in monocular rivalry [7], [10], [11], [16], [23], [24]. Alternatively monocular rivalry might stimulate similar mechanisms at lower levels of the visual pathway, but would not share the same higher-level activation patterns. Some previous studies have provided evidence that monocular rivalry may be mediated by opponent mechanisms at a low level of the visual hierarchy, such as V1 [27]–[30].

In psychophysical experiments, O'Shea et al. (2009) recently described several intriguing similarities between binocular and monocular rivalry, suggesting that common cortical mechanisms may underlie both forms of bistability [23]; however this has not been tested in a functional magnetic resonance imaging (fMRI) paradigm. Furthermore, we hypothesized that there may be an interaction of binocular and monocular rivalry with stimulus contrast, because binocular rivalry shows greater perceptual suppression than monocular rivalry, particularly at higher contrasts [23]. We anticipated that the effects of perceptual suppression would be evident in a lower BOLD signal for binocular rivalry compared with monocular rivalry in early visual areas, such as V1, V2 or V3 [3], [32]. It is not clear whether the effects of suppression would be limited to V1 or would include V2 and V3, because it has been proposed that the effects of suppression increase with each level of the visual hierarchy [10], [11], [23], [24], [27]. No previous fMRI study of rivalry has varied contrast systematically in order to study these effects.

In the present experiments we make a direct comparison of binocular and monocular rivalry in an fMRI paradigm, in which subjects performed a task to measure alternation rates. We used orthogonal gratings presented either dichoptically for binocular rivalry (different image in each eye) or monoptically (same image in each eye) for monocular rivalry. Coloured stimuli were used in order to enhance the percept of monocular rivalry [33], [34]. We also used replay conditions, in which the entire stimulus was physically changed between the two possible percepts, using the identical temporal sequences reported during rivalry with button presses earlier in the scans [3], [12], [18]–[20]. The comparison of rivalry with the replay condition allowed us to isolate the neural substrates specific to the perception of rivalry.

## Methods

### Ethics Statement

The subjects provided informed written consent and were remunerated for their time. The experiments were approved by the Research Ethics Board (REB) of McGill University (Protocol NEU-08-03).

### Subjects

Two authors (AB, SJ) and four subjects who were naïve as to the hypotheses of the study participated in all experiments. The subjects (which included two women) were university students or postdoctoral fellows (age range 20–40, mean  = 28). All were right-handed and had normal or corrected-to-normal acuity and stereoacuity thresholds better than 30 s arc, measured using the Titmus stereo test (Stereo Optical Co., Chicago, IL).

### Display

All stimuli were presented on a MacBook Pro Laptop (Intel Core 2 Duo) Macintosh computer with 1024×768 resolution, 120 Hz refresh rate with 8 bit/pixel greyscale, which was gamma-corrected using a colour look-up table. After calibration, the stimulus had a mean luminance of 30 cd/m^2^ and peak luminance of 60 cd/m^2^. Stimuli were generated and displayed using Matlab (2007b) and Psychtoolbox Version 3 (PTB-3) [35], [36] software and a Matrox (Dual Head 2Go Analogue Edition) splitter graphics card. Dual LCD (InFocus LP 540) projectors and linear polarizers were used for dichoptic projection [37]. The subjects wore linear polarizers with complementary polarization on their eyepieces. The stimuli were back-projected from the LCD projectors onto a screen at a viewing distance of 134 cm. The same display apparatus were used both for fMRI scan sessions and psychophysical sessions, including the same viewing distances. In the case of fMRI, the screen was placed at the rear end of the MR scanner bore and subjects viewed stimuli through a mirror attached to the head coil. Throughout the experiments, each stimulus was projected through an opaque rectangular aperture (5.1 deg height x 3.7 deg width), to minimize edge disparities. This relatively large stimulus size was used to enhance the fMRI signal. Pilot testing carried out before the main experiments validated the choice of stimulus size, since subjects perceived a composite image (in which neither grating stimulus was exclusively visible over at least two thirds of the image) for less than 10% of the periods of binocular or monocular rivalry alternations.

#### Oblique left/right grating stimuli

The oblique left/right gratings were sinusoidal 1.5 cycles per deg (cpd) grating stimuli converted to two-tone (i.e. square wave) images [38] (Figure 1). Orthogonal orientations were used (45, -45 deg; 60, -30 deg; 75, -15 deg).

The grating stimuli were coloured red/green (CIE red: x = 0.377, y = 0.363; green: x = 0.350, y = 0.394) in order to enhance the perception of monocular rivalry [33], [34], and were presented on a yellow background (CIE: x = 0.362, y = 0.377). In all of the psychophysical tests and fMRI scans, colour counterbalanced versions of the stimuli were used (i.e. each stimulus component could be red or green). Red-green isoluminance was confirmed for each subject individually using a minimum motion technique [39] for gratings viewed binocularly with the same mean luminance and chromaticity as in the main experiment; none of the subjects required different luminances for the red and green gratings.

### Psychophysical tests

Psychophysical testing was carried out before the fMRI scanning sessions in order to ensure that appropriate stimulus parameters were used.

#### Alternation rates

Alternation rates were measured for binocular or monocular rivalry with the left/right oblique grating stimuli. Subjects reported perceptual alternations continuously over 90 s trials. For binocular rivalry, subjects pressed one key when the left oblique stimulus predominated (over at least two-thirds of the stimulus), or another key when it was not visible. The subjects were tested a second time with the meaning of the keys reversed. Before each trial, the subject was reminded which key corresponded to which stimulus. For monocular rivalry, subjects pressed the key when each stimulus component appeared to be at least twice as clear as the other, or was exclusively visible over at least two-thirds of the stimulus (the same criterion for visibility as used by O'Shea et al., 2009). Contrasts of 4.5%, 9%, 18% and 36% were used, and subjects were tested twice at each condition.

#### Suppression test (visibility)

The binocular rivalry stimulus was presented on the left side of the screen (reference condition). An image on the right side of the screen (test condition) displayed only one stimulus component, which was one of the stereo half-images in the binocular rivalry stimulus (i.e. single grating only). The subject adjusted the contrast of the test image until it matched the apparent contrast of that component in the binocular rivalry (reference image) when that component was maximally suppressed during alternations. Because some binocular rivalry alternations could be incomplete, subjects were instructed to match the contrast during alternations in which most of the image was suppressed. The results provided an estimate of suppression during binocular rivalry alternations. A similar version of the test was also used with the monocular rivalry stimulus (i.e. grating composite) as the reference image and the test image showed one component (i.e. single grating). The subjects adjusted the contrast of the test image until it matched the apparent contrast in the reference image when that component appeared to have the lowest contrast during monocular rivalry alternations. Reference contrasts of 4.5%, 9%, 18% and 36% were used, and subjects were tested twice at each condition. It should be noted that our test of suppression emphasizes visibility, as opposed to measures of sensitivity in which the detection of a test probe is made during the dominance and suppression phases of rivalry, according to distinctions made by certain investigators [24].

### Functional Magnetic Resonance Imaging

#### Acquisition of fMRI data

All images were acquired using a 3T MR scanner (Siemens, Trio, Germany) at the Montreal Neurological Institute, with a 32-channel head coil (20-channels for retinotopic mapping). Functional whole brain images were acquired using a T2*-weighted gradient echo, echo-planar imaging sequence (38 slices, repetition time (TR) 2500 ms, echo time (TE) 30 ms, FOV 192, voxel size 3×3×3 mm). Functional images for retinotopic mapping were acquired with a T2*-weighted sequence, with slices oriented perpendicular to the calcarine sulcus (28 slices, repetition time (TR) 2000 ms, echo time (TE) 30 ms, FOV 128, voxel size 4×4×4 mm). Anatomical images were acquired by using a T1-weighted magnetization-prepared rapid gradient-echo (MP-RAGE) sequence optimized for contrast between grey and white matter (176 slices, repetition time (TR) 2,300 ms, echo time (TE) 2.98 ms, FOV 256, voxel size 1×1×1 mm).

#### Monocular/binocular rivalry scans

A block design was used composed of 30 s stimulus blocks. The first half of the scan consisted of blocks alternating between binocular and monocular rivalry in ascending contrasts (9%, 18%, 36%). These blocks were presented in the same order in all scans, to minimize the effects of adaptation or afterimages. The alternating order of rivalry condition was also the same in all scans. Subjects used a button box to report when their dominant percept switched to that of a left oblique or right oblique grating (following the procedure described above). Unlike some previous experiments on binocular rivalry, we did not design the experiment to directly compare short periods when the left oblique was dominant to those when the right oblique was dominant. Instead, we chose to isolate the cortical areas recruited during longer blocks of rivalrous viewing [20], [40].

During the second half of each scan, a non-rivalrous replay condition was presented in the same order as in the first half of the scan. In the replay conditions the entire stimulus physically changed between the two possible percepts (i.e. left or right oblique grating) which were perceived during alternations, duplicating the exact temporal sequence of the button presses from the first half of the scan. Following the methods used previously for binocular rivalry replay [20], the entire left or right oblique grating stimulus was shown to both eyes, and no composite images were shown. The contrast was modulated using a boxcar function (i.e. images were shown immediately at full contrast). During this second part of the scan, the subject used the button box to report when the stimulus switched between the replayed conditions. Finally, for all scans, the first and last block of each scan were blank baseline blocks. In total, each scan included 14 blocks (6 blocks of rivalry, 6 blocks of replay and 2 blank baseline conditions). Each subject participated in four scans with the grating stimuli. The scans were counterbalanced for colour (e.g. the colours of the left and right oblique gratings were interchanged). The left/right oblique gratings were presented at three different orthogonal orientations (45, -45 deg; 60, -30 deg; 75, -15 deg) to minimize the effects of adaptation. The orientations of the gratings were constant within a block, and were presented in the same order in all scans.

#### Retinotopic mapping and the localization of MT+

Retinotopic mapping was carried out in a separate session. The stimuli for retinotopic mapping consisted of high contrast, chromatic, flickering checkerboard patterns of two specific types. A rotating wedge stimulus swept through polar angles, and an expanding/contracting ring stimulus mapped eccentricity. There were four acquisition scans for each subject; eccentricity mapping (fovea to periphery and vice versa), and polar mapping (clockwise and counter-clockwise). The polar mapping scans consisted of 8 cycles (full hemifield rotation of both wedges), lasting a total of 512 s. The eccentricity mapping scans consisted of 8 cycles of expanding or contracting rings, lasting a total of 512 s. Both stimuli attempted to compensate for the cortical magnification factor by increasing in size as they approached the periphery. The eccentricity stimuli traversed space with a logarithmic transformation. A central fixation marker was present at all times, and subjects were required to perform a task monitoring the orientation of the fixation marker to aid fixation stability. These retinotopic mapping scans were used to define foveal regions-of-interest for V1, V2 and V3, defined as the region of occipital pole activated in the central 2.9 deg of visual angle. Area V3A was also defined using these scans. In addition, subjects performed two scans of MT+ localization (256 s) consisting of eight 16 s epochs of low contrast stationary rings and eight 16 s epochs of moving rings [41].

#### Data analysis

We used the BrainVoyager QX analysis package, version 1.10.4.1250 (Brain Innovations, Maastricht, The Netherlands) for most functional data analyses as well as for the creation of inflated and flattened cortical representations. The freely available Freesurfer analysis package, version v4.5.0 (http://surfer.nmr.mgh.harvard.edu/), was found to be better for retinotopic mapping data analysis on the reconstructed inflated brain, and the identified retinotopic areas were transferred to BrainVoyager using anatomical landmarks.

The anatomical and functional scans were analyzed in BrainVoyager using a standard processing sequence, described as follows. The anatomical scans were used to create surface reconstructions of each subject's cerebral cortex. The computed cortical surface representation was inflated and then flattened. Each subject's reconstructed folded cortical representation was normalized to spherical coordinate space and aligned to a target brain (chosen as an individual subject) using cortex-based alignment. The cortex-based alignment was performed in order to obtain a good match between corresponding brain regions for the group-level statistical data analysis. Before analysis of the functional scans, the first two volumes of every scan were discarded. All functional images were subjected to a standard set of preprocessing steps: (1) motion correction; (2) slice timing correction; (3) linear trend removal using a high-pass filter; (4) transformation of the functional data into Talairach coordinate space [42]; and (5) coregistration to anatomical images. A voxel-by-voxel, fixed effects general linear model (GLM) was used for analysis, with all of the stimulus conditions as predictors (i.e. rivalry and replay conditions at all contrasts, and baseline). The functional results were then viewed on an individual's cortical surface, producing maps of statistical significance (t-tests with a false discovery rate of p<0.05, and corrected for multiple comparisons). In addition, we separately analyzed the BOLD signal changes within regions of interest (retinotopic areas, MT+), using a fixed effects GLM analysis.

#### Additional regions of interest

Two frontal regions of interest, Brodmann's areas 44 (pars opercularis of the inferior frontal gyrus), and 47 (ventral orbital frontal cortex), as well as inferior parietal cortex were defined anatomically for each subject using the Talairach brain atlas in BrainVoyager, which can be used to visualize Brodmann's areas [42] on individual subjects's brains.

## Results

### Psychophysics

**Figure 2:**
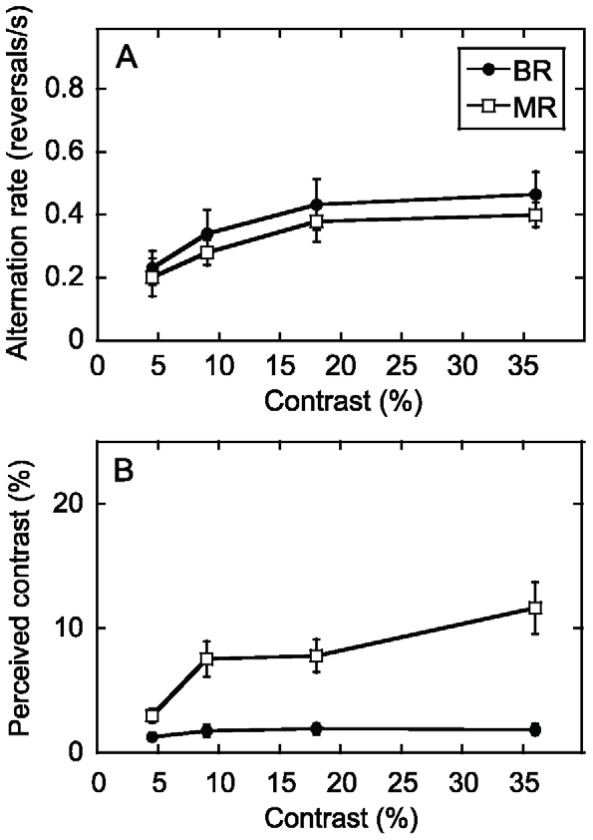
Psychophysical data averaged across all six subjects. (A) Alternation rates for binocular or monocular rivalry with grating stimuli. There was a slight tendency for alternations to be slower for monocular than binocular rivalry, but this was not statistically significant. (B) Data from the suppression test for binocular or monocular rivalry with grating stimuli. There was a greater change in visibility with alternations for binocular than monocular rivalry, especially at higher contrasts. Error bars are ±1 s.e.

Psychophysical testing was carried out before the fMRI sessions in order to determine the most appropriate contrasts for the binocular and monocular rivalry stimuli (Figure 2). There was a slight tendency for alternations in monocular rivalry to be slower than those for binocular rivalry, but both had approximately the same dependence on contrast, increasing as contrast was increased, and reaching a plateau at high contrasts. A two-way repeated measures ANOVA revealed that the effect of contrast was significant (F(3,15) = 6.95, p<0.05), but not rivalry condition (binocular/monocular) (p>0.05) nor the interaction between these factors (p>0.05).

The results of the contrast adjustment task provided a measure of the apparent contrast of a stimulus component in either binocular or monocular rivalry when it was maximally suppressed, thus providing a measure of suppression (Figure 2B). The alternations in binocular rivalry were accompanied by much greater suppression than monocular rivalry, associated with a much greater reduction in visibility of the suppressed pattern. This was particularly evident at the highest contrast at which the suppression in binocular rivalry was close to complete, while the monocular rivalry alternations were hard to perceive [23]. A two-way repeated measures ANOVA revealed that the effect of contrast (F(3,15) = 25.9, p<0.05) and rivalry condition (F(1,5) = 30.4, p<0.05) as well as the interaction between these factors (F(1,5) = 23.6, p<0.05) were all significant. The interaction of rivalry condition with contrast occurred because the measured suppression decreased with contrast for monocular rivalry but not for binocular rivalry. On the basis of these psychophysical results, we judged it appropriate to use a range of low to intermediate contrasts (9%, 18%, 36%) for fMRI in order to dissociate binocular and monocular rivalry and test the interaction with contrast.

### FMRI Comparison of Monocular and Binocular Rivalry to Baseline

**Figure 3:**
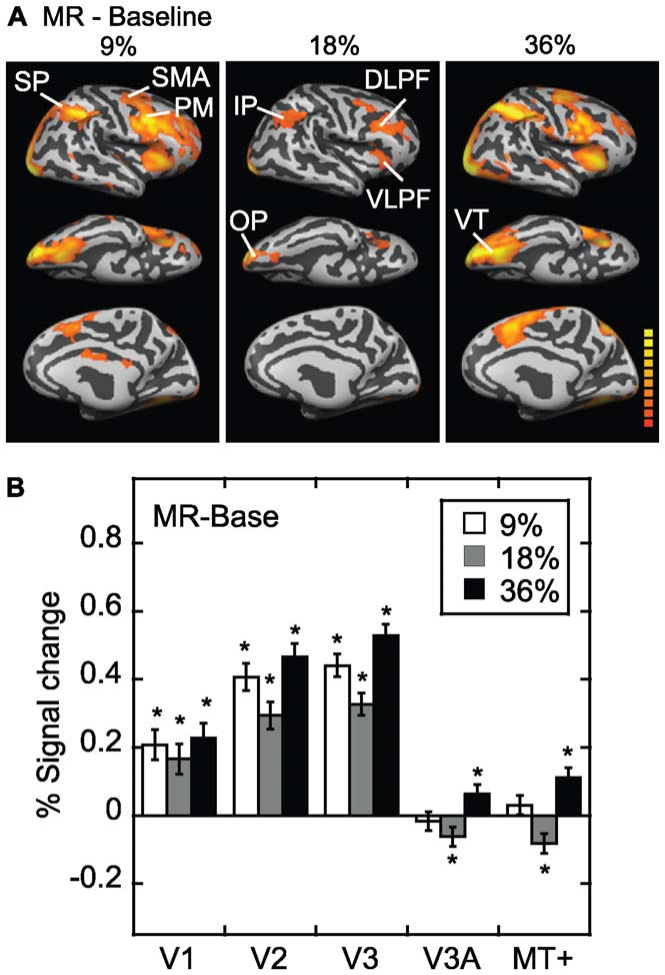
Monocular rivalry minus blank baseline. (A) Activation for monocular rivalry (MR) with grating stimuli above the blank baseline condition at the three contrasts (9%, 18%, 36%). The lateral, ventral and medial views of the inflated brain are shown (right hemisphere only). Colour scale on this and subsequent Figures indicates statistically significant results ranging from t = 2.35 to 8.00 (orange-yellow) (FDR, p<0.05). Monocular rivalry showed a U-shaped function of activation as a function of contrast; there was higher activation in a number of different areas at 9% and 36%. Abbreviations: dorsolateral prefrontal cortex (DLPF); inferior parietal cortex (IP); occipital pole (OP); premotor cortex (PM); superior parietal cortex (SP); supplementary motor area (SMA); ventral temporal (VT); ventrolateral prefrontal cortex (VLPF). (B) Region of interest analysis for monocular rivalry, in terms of percent signal change above the blank baseline condition (average of six subjects). The results are shown for gratings at the three contrasts (9%, 18%, 36%). The analysis for areas V1, V2 and V3 was carried out only in the foveal part of each area (0–2.9 deg eccentricity). Generally, the results did not differ between the left and right hemisphere, and have been averaged. Statistically significant results (p<0.05) in this and subsequent Figures are labeled with an asterisk. As in panel (A), monocular rivalry showed a U-shaped function of activation as a function of contrast in many areas, particularly V2 and V3.

The whole-brain pattern of activation obtained for monocular pattern rivalry compared to baseline, averaged for all six subjects, is shown in Figure 3A. That is, 30 s epochs of rivalry are compared to a blank screen. A network of regions is recruited during the experience of rivalry that includes the occipital pole, ventral temporal areas, inferior parietal cortex, and dorsal and ventral prefrontal cortex. In general, this network showed a U-shaped function of activation as a function of contrast, i.e. higher activity for most areas at 9% and 36%. At the highest and lowest contrasts (36% and 9%), some additional areas were activated which were not (significantly) activated at the middle contrast, for example, superior parietal cortex, supplementary motor area and premotor cortex. The U-shaped function in the activation is consistent with predictions of current models of binocular rivalry [7], [8], [10], [11], [16]. The increase in activation at higher contrasts can be explained due to neuronal response gain [11], [43]. The increase in activation at the lowest contrast could be explained as a form of disinhibition, assuming that inhibitory neurons would only be weakly stimulated, resulting in slow alternations (see Discussion) [7], [8], [10], [11], [16].

Figure 3B shows the results of the corresponding visual area region of interest analysis, which shows robust activity in areas V1 – V3, but much less activation of V3A and MT+. It can be seen that monocular rivalry also trended towards a U-shaped function of activation as a function of contrast in these retinotopic areas. However, the quadratic trend in one-way ANOVA for effect of contrast in each visual area was generally not significant (p>0.05).

**Figure 4:**
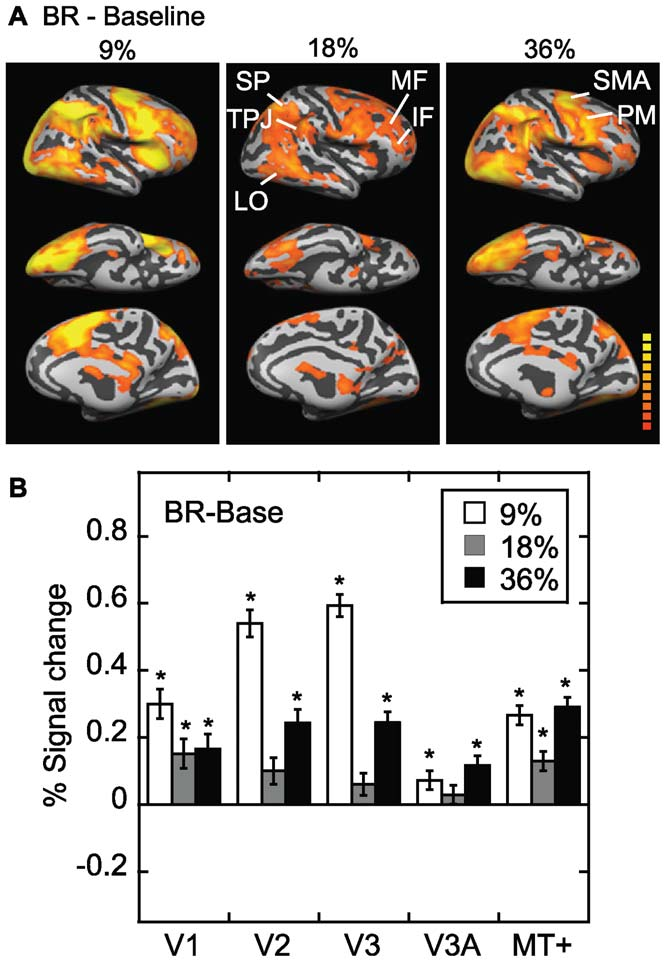
Binocular rivalry minus blank baseline. Figure follows the same format as Figure 3, but results are for binocular rivalry (BR) minus the blank baseline condition. (A) As with monocular rivalry, binocular rivalry showed a U-shaped function of activation as a function of contrast; there was higher activation in a number of different areas at 9% and 36%. Abbreviations: inferior frontal cortex (IF); lateral occipital cortex (LO); middle frontal cortex (MF); premotor cortex (PM); superior parietal cortex (SP); supplementary motor area (SMA); temporoparietal junction (TPJ). (B) The region of interest analysis also confirmed that binocular rivalry showed a U-shaped function of activation as a function of contrast in many areas, particularly V2 and V3.

Figure 4A shows the network of regions recruited for epochs of binocular rivalry compared to blank screen for all six subjects, for all stimulus contrasts. It is apparent that the activation for binocular rivalry was generally greater and more widespread than for monocular rivalry. The activated areas for binocular rivalry also included additional parts of the inferior parietal cortex near the temporoparietal junction, as well as superior parietal cortex, lateral occipital regions (including MT+), middle and inferior frontal cortex, premotor cortex and supplementary motor area. The overall pattern of activation for binocular rivalry, including frontoparietal areas, is consistent with previous studies [17], [19], [44]. For the retinotopic regions of interest shown in Figure 4B, all areas showed activation. The U-shaped function with stimulus contrast is again apparent. The one-way ANOVA for the effect of contrast in V2 and V3 showed significant linear effects (F(1,5) = 60.1, p = 0.001; F (1,5)  = 47.5, p = 0.001, respectively) as well as quadratic effects that were significant for V3 but just missed significance in V2 (F (1,5)  = 8.81, p = 0.03; F (1,5)  = 5.08, p = 0.07, respectively).

When interpreting the greater activation for binocular than monocular rivalry it is appropriate to consider the rivalry alternation rates that subjects experienced in the scanner (Table 1). A one-way repeated measures ANOVA of the key presses data revealed that the effect of contrast was significant (F (2,10)  = 9.04, p<0.05) but not rivalry condition (binocular/monocular) (p>0.05), nor the interaction between these factors (p>0.05). So, although rivalry rate increased with contrast as expected, monocular and binocular rivalry did not differ at the contrasts used here. For example, at 18% contrast, the rate for binocular rivalry was slightly above that for monocular rivalry (0.412 vs. 0.402, equivalent to 12.4 vs. 12.1 key presses in a 30 s block) but was not significantly different.

**Table 1:** Alternation rates (reversals/s) during the scan session (averaged across six subjects), for binocular or monocular rivalry at the three contrasts (9%, 18%, 36%).

Binocular Rivalry	
9%	0.378
18%	0.412
36%	0.456
Monocular Rivalry	
9%	0.328
18%	0.402
36%	0.448

**Figure 5:**
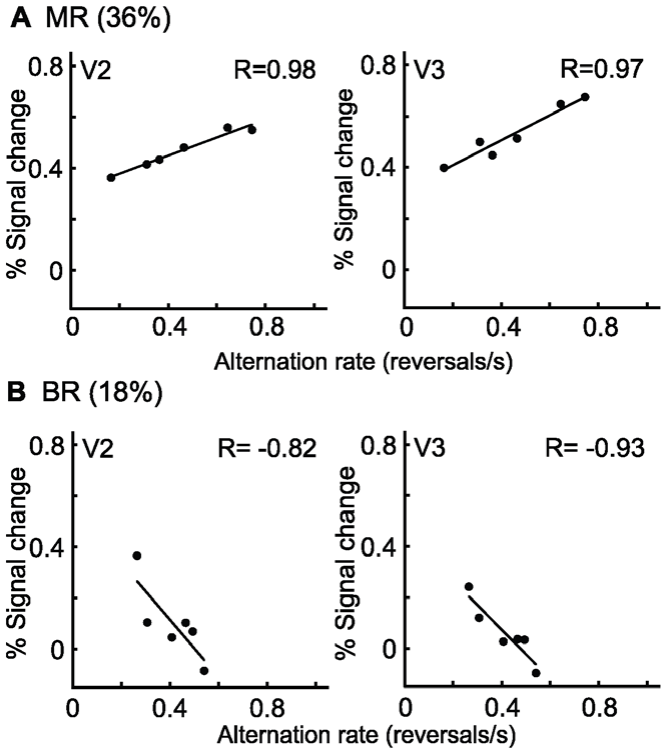
Correlations between activation for (A) monocular rivalry or (B) binocular rivalry and alternation rates. Correlations were performed between activation levels (% signal change minus baseline blank condition) and alternation rates for the six subjects obtained during the fMRI scan sessions. The correlations shown for monocular rivalry are for 36% contrast gratings, while the correlations for binocular rivalry are for 18% contrast gratings. Correlations are shown for areas V2 and V3 (average of left and right hemisphere), and in all four cases were statistically significant with correlation coefficients (r-values) of 0.82 and greater (p<0.05). For monocular rivalry, the activation levels increased with faster alternations, while the opposite effect occurred with binocular rivalry.

In addition to the analysis of mean fMRI signal change in Figures 3B and 4B, and mean alternation rates in Table 1, correlation analyses were carried out in order to determine whether the activation levels were correlated with the alternation rates for the six individual subjects. The results of the analyses for the retinotopic visual areas are shown in Table 2, and four of the significant correlations are shown in Figure 5. The activation for both monocular and binocular rivalry in areas V2 and V3 was significantly correlated with alternation rates, but remarkably the correlations were in opposite directions. For monocular rivalry, the activation was generally higher in subjects with faster alternations (i.e. positive correlation), and was significant in V2 and V3 at the highest contrast. There was also a strong positive correlation in V3A. These correlations for monocular rivalry may be related to neural response gain and alternation rates [11], [43]. In contrast, for binocular rivalry, the activation actually decreased for the subjects with faster alternations (i.e. negative correlation), but this was generally limited to the middle contrast (18%). This effect was significant in V2 and V3, and the negative correlations approached significance in V1. We speculate that for binocular rivalry the negative correlations occurred because at this middle contrast where the mean BOLD signal was low (bottom of U-shaped function, see Figure 4) some individual subjects with slower alternation rates had crossed into the pattern associated with low contrast (left side of U-shaped function). Hence, they entered the regime in which disinhibition at low contrast is linked to higher BOLD levels.

**Table 2:** Correlations between binocular or monocular rivalry activation (% signal change minus baseline blank condition) and alternation rates for the six subjects obtained during the fMRI scan sessions.

	Binocular Rivalry			Monocular Rivalry		
Visual Area	9%	18%	36%	9%	18%	36%
V1 LH	−0.75	−0.69	−0.22	−0.21	−0.48	−0.67
V1 RH	−0.28	−0.69	+0.066	−0.08	−0.08	−0.29
V2	+0.14	−**0.82**	+0.17	+0.69	+0.69	**+0.98**
V3	+0.25	−**0.93**	+0.032	+0.58	+0.58	**+0.97**
V3A	+0.36	−0.014	+0.026	**+0.81**	**+0.81**	+0.52
MT+	+0.55	+0.30	+0.26	+0.52	+0.39	+0.49

The Pearson correlation coefficients (r-values) are shown with positive or negative values, to indicate that activation levels increased (positive) or decreased (negative) with faster alternation rates. The correlation coefficients are shown for V1 (left hemisphere), V1 (right hemisphere), and other areas averaged for the left and right hemisphere (V2, V3, V3A, MT+). The statistically significant (p<0.05) correlations are shown in bold typeface.

In order to assess how widespread these significant correlations might be in the whole brain network subserving rivalry, exploratory correlation analyses were extended to selected parietal and frontal sites. Brodmann's areas 44 (pars opercularis of the inferior frontal gyrus), Brodmann's area 47 (ventral orbital frontal cortex), and inferior parietal cortex were chosen as areas that had been activated for both binocular and monocular rivalry (Figures 3, 4). None of the correlations for these non-visual regions of interest were significant, and are thus not reported.

### FMRI Comparison between Monocular and Binocular Rivalry

**Figure 6:**
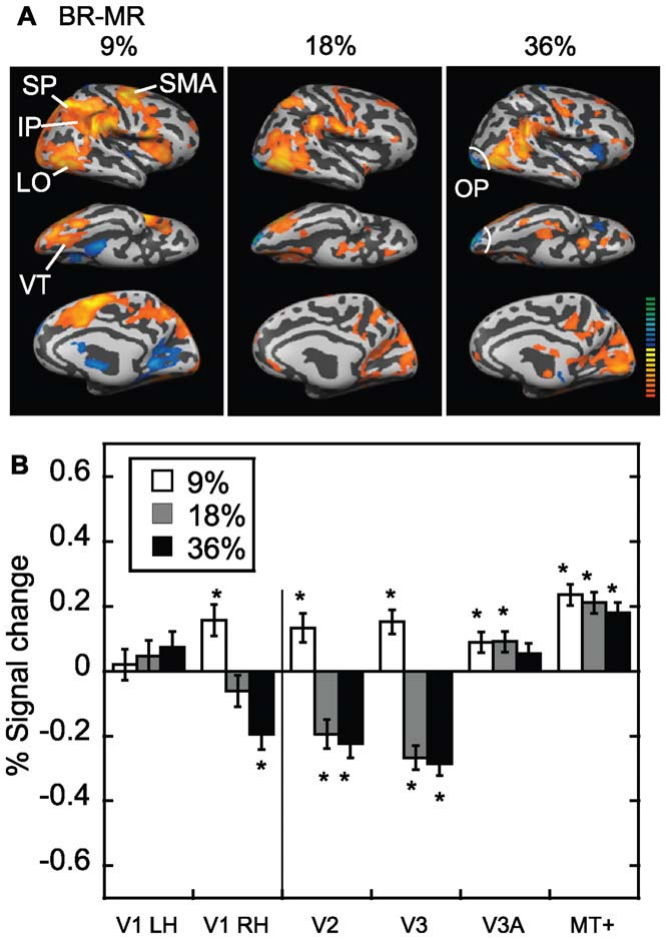
Binocular rivalry minus monocular rivalry. (A) Areas in which the activation for binocular rivalry exceeded that for monocular rivalry, shown for the three contrasts (9%, 18% and 36%). There was greater activation for binocular rivalry in occipital pole regions at the lowest contrast, but this reversed at higher contrasts. Note that the occipital pole (OP) is circled in white on a lateral and ventral view. Generally there was greater activation for binocular rivalry in superior parietal cortex (SP), inferior parietal cortex (IP) close to the temporoparietal junction, supplementary motor area (SMA), ventral temporal areas (VT) and lateral occipital (LO) areas including MT+ and lateral occipital complex. (B) Region of interest analysis. Binocular rivalry minus monocular rivalry in percent signal change (average of six subjects). The analysis for V1, V2 and V3 was carried out only in the foveal part of each area (0–2.9 deg eccentricity). Generally, the results did not differ between the left and right hemisphere, and have been averaged, except for area V1, for which the results are shown separately. There was greater activation for binocular rivalry in areas V2 and V3 at the lowest contrast, but this reversed at higher contrasts.

Figure 6A shows explicitly the differences between monocular and binocular rivalry at all three contrasts. When directly compared, greater activity is seen for binocular rivalry in superior and inferior parietal cortex (close to the temporoparietal junction), lateral occipital cortex, and ventral temporal areas. In addition to MT+, the lateral occipital region also included lateral occipital complex (LOC), as it matches the Talairach coordinates published for this area, for example 42.8±2.7, −72.7±8, and −18.2±9.8 [45]. Also of interest is the activity seen in the occipital pole region of cortex. It can be seen that for low (9%) contrast, the activation for binocular rivalry exceeded monocular rivalry in the occipital pole, but the effect reversed at the higher contrasts (small region shown in blue).

The corresponding region of interest analysis confirmed that the interaction with contrast in the occipital pole included early visual areas V1 (right hemisphere), V2 and V3 (Figure 6B). The results were plotted separately for left and right V1 because a significant laterality effect was evoked. We suggest that the psychophysics shown in Figure 2 can explain these results at higher contrasts. There was clearly greater suppression for binocular rivalry than monocular rivalry, particularly at the higher contrasts, which might be expected to lower the BOLD response. By comparison, a very different pattern of results can be seen for areas V3A and MT+. These areas were selective for binocular rivalry over monocular rivalry for all contrasts, which accords with previous studies showing that these regions are important in binocular integration for stereoscopic depth perception [17], [46], [47].

**Figure 7:**
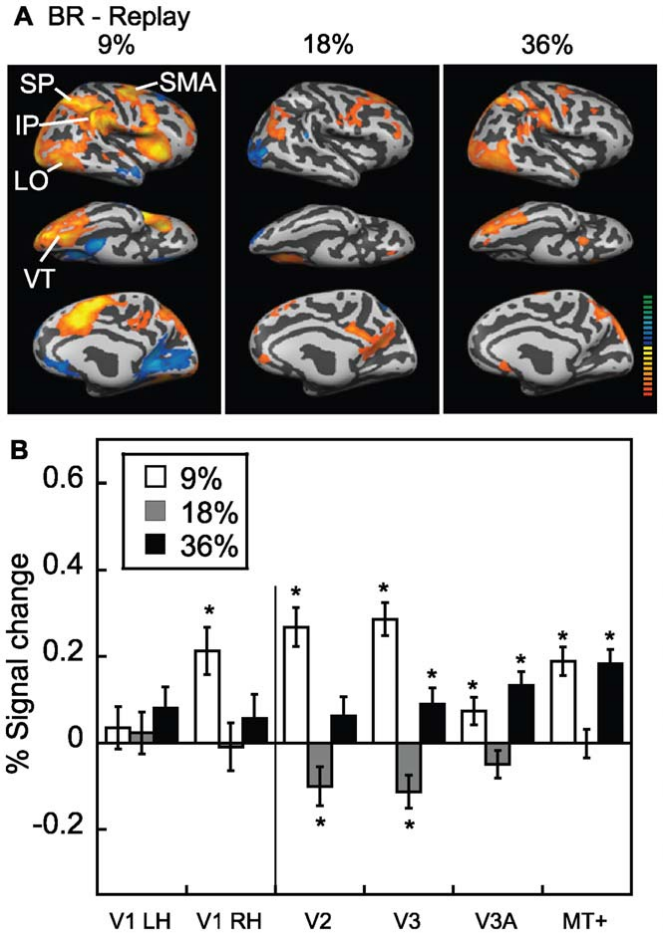
Binocular rivalry minus replay. (A) Figure follows the same format as Figure 6, but shows areas in which the activation for binocular rivalry exceeded rivalry replay. There was greater activation for binocular rivalry in superior (SP) and inferior parietal (IP) cortex, supplementary motor area (SMA), ventral temporal (VT) areas and lateral occipital (LO) areas, including MT+ and lateral occipital complex. (B) Region of interest analysis, as in Figure 6, but for the subtraction of binocular rivalry minus replay condition in percent signal change. With the replay condition subtracted, binocular rivalry continued to show a U-shaped function of activation as a function of contrast, with higher activation at 9% and 36%.

Finally, Figure 7A shows the areas in which binocular rivalry exceeded the replay condition, which was precisely matched for the temporal sequence of images, alternation rates, and button presses. Thus, this important subtraction serves to isolate rivalry-related perceptual processing (e.g. endogenously generated competition between perceptual alternatives). With the replay condition subtracted, binocular rivalry continued to show a U-shaped function of activation as a function of contrast (i.e. higher activation in many areas at 9% and 36%). The region of interest analysis (Figure 7B) further confirmed that the activation for binocular rivalry was above the replay condition at the lowest and highest contrast in areas V3, V3A and MT+. Binocular rivalry was also above the replay condition at the lowest contrast in V1 (right hemisphere) and V2. At the middle contrast, the activation for the replay condition sometimes exceeded binocular rivalry (V2, V3). All these results indicate that the activation for the replay condition did not show a U-shaped function of contrast but tended to grow monotonically or was constant as a function of contrast. Hence the U-shaped function is likely related to the perception of rivalry per se rather than to stimulus or response features. We might expect from models of rivalry that inhibitory interocular interactions, as well as neuronal adaptation and response gain are all factors that may affect binocular rivalry alternations and contribute to the fMRI BOLD response [7], [8], [10], [11], [16], [43].

A two-way repeated measures ANOVA for V1 (carried out separately for the left and right hemisphere) was not significant for any of the main effects of contrast or stimulus condition (rivalry vs. replay) (with either a linear or quadratic trend) or interactions (p>0.05). A similar analysis for V2 revealed a significant effect of contrast (F (2,10)  = 34.1, p<0.05), but not stimulus condition (rivalry versus replay) (p>0.05). The interaction between stimulus condition and contrast (F (2,10)  = 14.7, p<0.05) was significant. The analysis for area V3 again revealed an effect of contrast (F (2,10)  = 34.1, p<0.05), but not stimulus condition (rivalry versus replay) (p>0.05), along with a significant interaction (F (2,10)  = 12.2, p<0.05). In these analyses, the significant interaction of stimulus condition with contrast indicated that whether the rivalry condition exceeded replay depended upon contrast.

## Discussion

In this study we directly compared binocular rivalry with monocular rivalry and a non-rivalrous replay control condition. All three conditions were well-matched in terms of visual stimulus input and the motor task response. Despite considerable overlap in the whole-brain network recruited for all the tasks, distinct differences were also found. The overall pattern of activation was more widespread for binocular than monocular rivalry, suggesting that binocular rivalry may differ qualitatively in terms of recruitment of areas previously identified for binocular combination (V3A, MT+) [46], [48], [49] or for competitive attentional demands (TPJ) [50]–[52]. The effects of perceptual suppression were evident in early visual areas (V1, V2 and V3), in which activation was greater for binocular rivalry at the lowest contrast, but this effect reversed at the higher contrasts. The comparison of binocular rivalry with the replay condition was particularly important in isolating the neural substrates for the perception of rivalry, and highlighted these same regions of activation. These results are compatible with aspects of either stimulus-based or eye-based rivalry models. However, the prominent role of extrastriate areas in differentiating binocular and monocular rivalry is not compatible with an exclusively eye-based model that resolves binocular rivalry in V1, and lends some support to models of stimulus-based rivalry.

### Comparison of Rivalry with Baseline

Rivalry is a complex neural process that involves the interplay between adaptive and inhibitory functions. Rivalry is generally believed to comprise oscillations in the dominance of two sets of neurons, the activation of which would be of equal and opposite strength over time. Depending on the model of rivalry, the sets of neurons could represent left eye versus right eye input [7], [8], [10], [11], [16], or the representation of one stimulus versus the other [18], [25]–[27]. Previous work suggests that both models could exist in the brain, presumably, but not necessarily, in different visual areas. In particular, data suggests that eye-based rivalry would be more prevalent in early visual areas such as V1 or the lateral geniculate nucleus, while stimulus-based rivalry would dominate in higher-tier areas like inferotemporal cortex in primates, or LOC or ventral temporal cortex in humans [12]–[15], [18].

For both types of rivalry, it might not be clear that we would expect any change in the total BOLD signal of these combined populations of neurons in any particular area during blocks of rivalry. For example, if rivalry were occurring in V1 between neurons activated by the left and right eyes, activations would oscillate between neurons activated by either eye. The total activation would hold constant during the experience of rivalry, and might even remain independent of the alternation rate.

However, we found that binocular rivalry showed a U-shaped function of activation as a function of contrast. Current models of binocular rivalry can in fact be used to explain this pattern [7], [8], [10], [11], [16]. Rivalry models include inhibitory neurons in addition to excitatory neurons to account for interocular inhibition and suppression. In addition, the contribution of inhibitory neurons would generally be expected to lower the BOLD signal [3], [12]–[16], [32], [53]–[55]. In the case of higher contrast stimuli, we expect the activation to increase due to an increasing neuronal response gain (which also leads to faster alternation rates) [7], [8], [10], [11], [16], [43]. Presumably, the contribution of excitatory neurons would dominate, explaining the increase from 18% to 36% contrast.

The increase in activation at the lowest contrast can possibly be explained as a form of disinhibition, assuming that the excitatory and inhibitory neurons have different thresholds [7], [8], [10], [11], [16]. It is generally thought that at low contrasts inhibitory neurons are not strongly activated, resulting in slower alternation rates. If contrast is lowered even further (usually below 15%), a transition to single vision occurs and stable plaids are perceived [56]. Here, because of the use of colour to enhance rivalry, fusion of the images did not occur and binocular rivalry was still readily perceived at the lowest contrast (9%). We speculate that the higher BOLD signal at 9% contrast might be due, at least in part, to a release from inhibition that accompanies slow alternation rates. This interpretation is further supported by considering the variability in rivalry alternation rates between subjects. In particular, the negative correlations between alternation rate and fMRI signal in V1, V2, and V3 that we observed for the binocular rivalry condition lend some support to the disinhibition interpretation. It is the subjects with the slowest alternation rates that had the highest fMRI signal.

Nevertheless, an alternative interpretation of higher activation at low contrast is highlighted by noting that the U-shaped function was also evident in the whole brain network of parietal-frontal areas. The high activation at 9% can also be explained assuming that greater attentional resources may be recruited when discriminating between low (but suprathreshold) contrast images [57], [58]. In addition, after the replay condition was subtracted from binocular rivalry there was still a large response at the lowest contrast, which might reflect the effects of attention. Nonetheless, the fact that the U-shaped function was preserved for binocular rivalry, even after the replay condition was subtracted, suggests that it is related to the mechanisms involved in rivalry per se. The same low contrast images were used for rivalry and replay, and should be equally difficult to discriminate. Future experiments would be required to fully disambiguate this issue. Using techniques with increased temporal resolution (e.g., EEG, MEG or TMS) would be complementary and might further isolate these excitatory and inhibitory factors.

A limitation of the experimental design was that we do not report a replay condition for monocular rivalry. Although there is an established method for replay for binocular rivalry that has been used in a number of previous studies [3], [12], [18]–[20], this does not exist for monocular rivalry. The monocular rivalry replay condition might arguably require plaids rather than gratings, with appropriate contrast changes, and additional experimentation to select contrasts. It is also problematic that the plaids themselves might undergo rivalry. This does constrain the current interpretation of the monocular rivalry results, since the comparison against baseline does not isolate rivalrous perceptual mechanisms. Finally, given that the experimental design used a fixed order of presentation of contrasts, orientations or rivalry type, and the psychophysical testing was carried out before the fMRI scans, an effect on the results cannot be ruled out.

### Comparison of Binocular and Monocular Rivalry

When binocular and monocular rivalry were directly compared, another interaction with stimulus contrast was found in V1, V2, and V3. In this case, binocular rivalry evoked greater activation than monocular rivalry for the low contrast images. However, at higher stimulus contrasts, where perceptual suppression was more complete for binocular than monocular rivalry, the response to binocular rivalry fell below that to monocular rivalry. This provides novel evidence that for blocks of rivalry (that were matched for alternation rate) in which subjects experienced a greater amount of perceptual suppression, the BOLD signal was reduced. This is consistent with and adds weight to a number of studies that show that the fMRI signal in V1–V3 is reduced when stimuli that remain on the retina are *perceptually* suppressed with reduced visibility [3], [12], [13], [32], [53], [54]. We note in passing that a right hemisphere bias for binocular rivalry in V1 (Figures 6 and 7), is consistent with previous studies [19].

It should be acknowledged that there are concerns in inferring the relationship of the fMRI signal to visual perceptual processing, given the limited temporal and spatial resolution. Previous studies have related the fMRI BOLD signal and electrophysiological recordings in early visual areas such as V1 during binocular rivalry alternations (or flash suppression, a related visual phenomenon) in order to determine whether these measures reflect perceptual suppression [54], [59]. The results in V1 indicate that the lower frequency bands of the local field potential (LFP) and the fMRI BOLD response showed decreases during perceptual suppression, whereas neuronal spiking or the higher frequency band of the LFP were unaffected [54]. The lower frequency LFP has also been found to be closely correlated with perceptual suppression in areas V2 and V4 [59]. In comparison, for physical modulations of the stimulus, all of the electrophysiological signals as well as the BOLD response were closely correlated to stimulus visibility. Hence these studies reveal that low frequency LFP and BOLD (possibly biased towards presynaptic signals) may selectively reflect perceptual suppression. Moreover, fMRI does not always correspond to spike-related measures, and should be interpreted accordingly [60]. A comparison of fMRI and electrophysiological results in area MT+ have also shown that these measures reveal somewhat divergent physiological processes but provide complementary information [61].

Finally, the results of the present study can be compared to two previous fMRI studies using similar stimuli. Lee and Blake (2002) found less activity for binocular rivalry (left and right-oblique grating stimuli) compared to a plaid shown to both eyes in early visual areas (V1, V2, V3, V4v). This suggested that the presence of suppression in rivalry reduced the fMRI signal, and is consistent with our results at higher contrasts (although perceptual suppression was not measured in that study). We note that another fMRI study did not find any significant differences between activation levels for plaid patterns and binocular rivalry stimuli [32]. However, their plaids were presented monocularly, in the periphery, in short stimulus blocks, and they used a demanding foveal task unrelated to the perception of rivalrous alternations. So, a number of differences in stimulus presentation and task could account for the discrepancies.

It is notable that lateral occipital cortical areas, including MT+ were selective for binocular over monocular rivalry at all contrasts. Activation of MT+ has not always been noted in previous fMRI studies of binocular rivalry [19], [20], but has been found in a number of studies of bistability [17], [44], [47], [62], [63]. However, in most of these studies the form of bistability involved motion; the only exceptions used a slant rivalry stimulus in which alternations occurred between a depth- and perspective-based percept [17], [47]. Thus MT+ might contribute generally to the network that mediates perceptual ambiguity and change detection, and it is possible that subjects may experience apparent motion with conscious visual perceptual changes during binocular rivalry alternations. However, it should not be ruled out that MT+ is involved specifically in binocular competition. A preference for binocular stimuli is not surprising given that MT+ contains an ordered map of binocular disparity [46] and is involved in binocular depth perception [48], [49].

The lateral occipital activation found in the present study for binocular rivalry likely included areas beyond MT+. The LO cortex has been implicated in the perception of binocular depth or shape defined by disparity [17], [47]–[49], [64]. The LO has also been shown to be involved in bistability in a slant rivalry paradigm with alternations between depth and perspective percepts [17] or in studies of bistability with the Necker cube [65]. An area adjacent to MT+ (area KO) has been found to be responsive to depth structure, from either disparity or motion cues [66], while another occipito-temporal region anterior to MT+ is activated by cyclopean stereomotion-in-depth [67].

In addition to the activation of visual areas presumed to be involved directly in competition between neural representations, there was also activity in frontoparietal areas that are often implicated in attention, and previously identified for binocular rivalry [17], [19], [20], [44]. We show here for the first time that the temporoparietal junction (TPJ) is an area activated more by binocular rivalry than monocular rivalry. It is of high interest that the TPJ is generally modulated by stimulus-driven attentional shifts to behaviourally relevant stimuli, such as the appearance of new objects at unattended locations, and unexpected events [50]–[52]. During binocular rivalry the TPJ would likely be activated with alternations as these are important novel perceptual events that reorient attention. It is possible that monocular rivalry does not activate the TPJ because there is no object identity change with alternations. Rather, the stimulus always appears to be a composite of two stimulus components, not a change between two distinct objects. In fact, monocular rivalry seems unique in being a form of bistability that does not (significantly) activate TPJ, unlike binocular rivalry [19], ambiguous figures [65], [68], [69], apparent motion [44], [62], structure from motion [63] or filling-in [70].

As mentioned in the introduction, there has been some suggestion that monocular rivalry might share similar mechanisms of stimulus-based rivalry with binocular rivalry, but lack any component of eye-based rivalry, accounting for greater suppression in binocular rivalry. The current results are partially in line with this suggestion. We did in fact observe a reduced BOLD signal in V1 (right), V2, and V3 for binocular rivalry at the higher contrasts where binocular rivalry shows greater suppression. However, the current results also highlighted that monocular rivalry lacks features of the pattern of higher-tier activation for binocular rivalry (i.e. MT+ and lateral occipital complex, and TPJ).

Monocular rivalry thus differs from comparable binocular conditions primarily in terms of greater activation at high contrast in early visual areas (i.e. V1, V2 and V3), and reduced activation at all contrasts in higher-tier levels of visual association cortex. We consider these results consistent with previous psychophysical studies that have come to the conclusion that monocular rivalry results from competition in early visual areas [28]–[31], with mechanisms similar to those involved in transparency. We have also observed that the perception of alternations can be reduced by violating Metelli's law [71].

In conclusion, these results provide a new comparison between two forms of bistability. The patterns of neural activation could clearly be related to the perception. In particular, the effects of greater perceptual suppression in binocular rivalry could be related to reduced activation of early visual cortical areas. The greater perceived change in stimulus features in binocular rivalry could be related to enhanced activation of cortical areas implicated in shifts of attention to novel objects. These forms of bistability provide an important probe of conscious visual perception, fluctuating as the stimulus remains constant, and pivotal in the seamless experience of natural vision.
